# Neovascularization: The Main Mechanism of MSCs in Ischemic Heart Disease Therapy

**DOI:** 10.3389/fcvm.2021.633300

**Published:** 2021-01-26

**Authors:** Weili Shi, Qiqi Xin, Rong Yuan, Yahui Yuan, Weihong Cong, Keji Chen

**Affiliations:** ^1^Laboratory of Cardiovascular Diseases, Xiyuan Hospital, China Academy of Chinese Medical Sciences, Beijing, China; ^2^National Clinical Research Center for Chinese Medicine Cardiology, Beijing, China

**Keywords:** mesenchymal stem cells, ischemic heart disease, neovascularization, angiogenesis, vasculogenesis

## Abstract

Mesenchymal stem cell (MSC) transplantation after myocardial infarction (MI) has been shown to effectively limit the infarct area in numerous clinical and preclinical studies. However, the primary mechanism associated with this activity in MSC transplantation therapy remains unclear. Blood supply is fundamental for the survival of myocardial tissue, and the formation of an efficient vascular network is a prerequisite for blood flow. The paracrine function of MSCs, which is throughout the neovascularization process, including MSC mobilization, migration, homing, adhesion and retention, regulates angiogenesis and vasculogenesis through existing endothelial cells (ECs) and endothelial progenitor cells (EPCs). Additionally, MSCs have the ability to differentiate into multiple cell lineages and can be mobilized and migrate to ischemic tissue to differentiate into ECs, pericytes and smooth muscle cells in some degree, which are necessary components of blood vessels. These characteristics of MSCs support the view that these cells improve ischemic myocardium through angiogenesis and vasculogenesis. In this review, the results of recent clinical and preclinical studies are discussed to illustrate the processes and mechanisms of neovascularization in ischemic heart disease.

## Introduction

Ischemic heart disease (IHD) is characterized by reduced blood supply to the heart and is the leading cause of death and disability worldwide. Long-term myocardial ischemia and acute massive myocardial infarction often result in decreased left ventricular function. Although the development of new drugs and the use of stent implantations have benefited numerous patients with coronary heart disease, some patients still have no effective treatment due to issues associated with diffuse coronary artery lesion, postoperative restenosis and heart failure after myocardial infarction (MI).

The foundation of IHD treatment is the reconstruction of vessels and the recovery of blood flow. Over the past decades, with the introduction of the concept of therapeutic angiogenesis, more and more studies have demonstrated that neovascularization can effectively improve the blood supply of ischemic myocardium. There are two primary mechanisms by which neovascularization occurs: vasculogenesis and angiogenesis. Vasculogenesis is the *in situ* assembly of endothelial progenitors into capillaries, while angiogenesis is a process through which new blood vessels form from pre-existing vessels through sprouting and intussusception (1). Cytokine-based therapeutic angiogenesis from the bench to clinical trials has been a major focus of medical research, and the efficacy of vascular endothelial growth factor (VEGF) blockers has led to the approval of anti-angiogenesis drugs for cancer and eye disease. Conversely, the use of angiogenesis factors, such as VEGF and basic fibroblast growth factor (bFGF), has been shown to promote notable increases in collateral vessel and myocardial perfusion in ischemic myocardium, reduced infarct size and improved cardiac function (2), demonstrating the theoretical and experimental promise of this approach in treating ischemic diseases. Unfortunately, despite the exciting results obtained using angiogenesis factors to treat IHD, gene therapy is also limited by its restricted efficacy and resistance (3). For example, VEGF also accelerates angiogenesis in atherosclerotic plaques and promotes plaque growth, which may eventually lead to plaque instability, while it promotes angiogenesis in ischemic tissue, an observation referred to as the famous Janus phenomenon (4). Angiogenesis greatly improves blood flow in myocardial ischemia, but the safety of growth factor-based angiogenesis therapy is an issue that remains to be overcome. Thus, how to avoid the risks associated with angiogenesis therapy is a problem that must be considered.

Stem cell-based therapies provide a promising new method for the formation of new blood vessels. MSCs have become the most promising seed cells for the treatment of IHD, with advantages of rapid self-renewal, multidifferentiation potential, and weak immunogenicity in autologous transplantation. Clinical and preclinical studies have shown that MSCs therapy effectively limits the infarcted area and improves heart function. However, the mechanisms associated with the activities of MSCs in IHD therapy remain controversial. We primarily attribute the cardiac protective effect of MSCs to their ability to promote neovascularization for the following two reasons. First, MSCs secrete soluble paracrine factors that contribute to angiogenesis and vasculogenesis. Second, MSCs are able to differentiate into ECs, pericytes and smooth muscle cells (SMCs), which form the foundation of vessels, processes that both participate in the protective ability of MSCs toward IHD. In this review, we focus on the mechanisms and clinical applications of MSCs in IHD therapy through neovascularization to provide reference for the application of stem cells in IHD.

## Comparison of MSCS From Different Sources

MSCs can be isolated from bone marrow, adipose tissue, umbilical cord blood, peripheral blood and almost every tissues in adults. Although MSCs can be harvested from different sources, regardless of their origin, they all have the capability of differentiating into adipocytes, osteoblasts and chondroblasts *in vitro* under specific conditions and can adhere to plastic under culture conditions. Furthermore, the surface of MSCs displays CD73, CD90, and CD105 but lack CD34, CD45, HLA-DR, CD14 or CD11b, CD79a or CD19. The International Society for Cell Therapy proposed the three criteria described above as identification standards for MSCs (5). Although MSCs from different sources share many of the same biological features, there are also some differences between distinct MSC populations. Bone marrow-derived MSCs (BMSCs), adipose-derived MSCs (AMSCs) and umbilical cord-derived MSCs (UCMSCs) are the most popular MSCs in clinical and preclinical experiments and trials, and some of their capabilities are compared below (Table 1).

**Table 1:** Comparison of MSCs from different sources.

	**BMSCs**	**AMSCs**	**UCMSCs**
**Differentiation capacity**			
Osteogenesis	++	+	+++
Chondrogenesis	++	+	+++
Adipogenesis	+	++	+++
Endothelial cells	+	+	+
Pericytes	+	+	+
Smooth muscle cells	+	+	+
Proliferation capacity	+	++	+++
Migration capacity	+++	+	++
Tube formation	+	+	+

### Differentiation Capacity

MSCs have the ability to differentiate into adipocytes, osteoblasts and chondroblasts. The amount of calcium deposits and sulfated proteoglycans stained by Alizarin red and Alcian blue, respectively were both higher in BMSCs than that observed in AMSCs, indicating that BMSCs have a higher capacity toward osteogenic and chondrogenic differentiation than AMSCs. While similar adipogenic differentiation potential was observed between these two types of cells (6), some studies have reported that AMSCs are more prone to adipogenic differentiation than BMSCs (7). Baksh et al. (8) observed that compared to BMSCs, UCMSCs underwent osteogenic differentiation more rapidly, exhibited higher alkaline phosphatase activity, and generated significantly more fat-containing cells when grown under adipogenic conditions by day 21. The differentiation ability of stem cells is affected by donor sex, age, isolation and culture conditions, etc. (9). Thus, which types of MSCs have a greater ability to differentiate into adipocytes, osteoblasts and chondroblasts remains disputed. In addition, MSCs also have the ability to differentiate into ECs, pericytes and SMCs, which are necessary components of blood vessels (10–13). Lu et al. (14) showed that MSCs from adipose tissue may have significantly greater ability to promote angiogenesis both *in vitro* and *in vivo* than UCMSCs and endometrial MSCs.

### Proliferation Capacity

MSCs from different tissue sources do not have the same proliferative ability *in vitro*. Choudhery et al. (15) observed that UCMSCs have higher population doublings than AMSCs (33.0 ± 1.5 vs. 25.8 ± 0.6), with the doubling time being longer for AMSCs (2.7 ± 0.03 days) than UCMSCs (2.0 ± 0.04 days). Moreover, after prolonged passaging (30 times), the proliferative ability of UCMSCs did not change significantly, while BMSCs showed decreased proliferation after 6 passages (8), indicating that UCMSCs have a stronger proliferative ability than BMMSCs and AMSCs. Under human platelet lysate-supplemented culture conditions, AMSCs were observed to have greater proliferative potential than BMSCs (6). Therefore, the proliferative ability of UCMSCs is the strongest, followed by AMSCs and BMSCs.

### Migration Capacity

MSCs play an important role in posttraumatic tissue repair and cell therapy, and their migration ability is a key factor affecting their therapeutic efficacy. The migration capacity of BMSCs and placenta-derived MSCs (PMSCs) was observed to be 5.9- and 3.2-fold higher than that of UCMSCs, respectively. These results were consistent with the observed levels of migration-enhancing proteins in UCMSCs, including cathepsin B, cathepsin D and prohibitin, which were significantly lower than those observed in BMSCs and PMSCs, while the levels of migration-inhibiting proteins such as plasminogen activator inhibitor-1 and manganese superoxide dismutase were higher (16). Vimentin also contributed to the higher migration capability of BMSCs than UCMSCs (17). In contrast, UCMSCs exhibited an enhanced migration capacity toward factors released by hepatocellular carcinoma compared with BMSCs (18).

### Capacity of MSCs to Promote Tube Formation of ECs

Tube formation is the last step in the formation of vessels and is necessary to supply blood for ischemia. Pill et al. (19) showed that AMSCs and BMSCs are both promising cell types to induce vascularization with ECs *in vitro* and are promising candidates to support *in vivo* vascularization. Nevertheless, Kim et al. (20) observed that conditioned medium from human AMSCs showed better tube formation-promoting effects than that from BMSCs *in vitro*, and AMSC group showed better recovery of blood flow than BMSC group in hindlimb ischemia model of nude mice. Furthermore, young AMSCs may have a higher tube formation capacity than old ones (21). UCMSCs are also capable of forming tubular networks (22). Du et al. (23) reported tube numbers of 11.65 ± 2.92, 0.91 ± 0.76 and 0.41 ± 0.20 for BMSC, AMSC and UMSC groups, respectively, indicating that BMSCs may have better angio-vasculogenic capacities than UMSCs and AMSCs. In contrast, Panepucci et al. (24) thought that UCMSCs would be more committed to angiogenesis and BMMSCs would be more committed to osteogenesis. Above all, there is still no consensus on which cell type has the greater capacity to promote tube formation.

## Paracrine Function of MSCs Throughout the Neovascularization Process

The mechanism of MSC therapy is still controversial, because few MSCs can be found in myocardium after injection *in vivo* study. Wang et al. (25) showed that most intravenously injected MSCs remain in the lungs and liver, with only a small portion reaching the myocardial tissue. Similarly, Uemura et al. (26) observed only a few GFP-labeled MSCs in the periinfarct myocardium. Even so, clinical and preclinical studies still indicated the cardiac function of ischemic heart was improved, and infarct size and the number of apoptotic cardiomyocytes were significantly reduced after MSCs intervention. Which suggested the efficacy of MSCs did not benefited from themselves in some degree.

Paracrine hypothesis was firstly advanced by Gnecchi et al. (27). They found genetically modified BMSCs overexpressing the Akt1 released paracrine factors that exert cytoprotective effects on cardiomyocytes exposed to hypoxia and limited infarct size and improved ventricular function (27, 28). Furthermore, high VEGF, bFGF, IGF-1 and SDF-1 expression in hypoxia-preconditioned MSCs medium was examined, the results of which indicated that the paracrine function of MSCs may play more important role than their differentiation ability (26). Recently, it has been reported that MSCs secreted a wide array of cytokines that exerted beneficial angiogenesis in ischemic tissue, including PDGF, thrombopoietin, and angiogenin (29, 30). These factors are all involved in the neovascularization process, including MSC mobilization, migration, homing, adhesion and retention, and the differentiation of ECs. Especially VEGF and bFGF, which both have high affinity toward heparin and participate in angiogenic processes such as migration and amplification of ECs, are also necessary substances to induce the transformation of stem cells into ECs (10, 31). MSCs overexpressing Akt and angiopoietin-1 showed higher Flk1 and Flt1 positivity and promoted intrinsic Flk1^+^ and Flt1^+^ cell mobilization into the infarcted heart (32). Huang et al. (33) observed that overexpression of miR-126 promoted the differentiation of MSCs toward ECs through activation of the PI3K/Akt and MAPK/ERK pathways and the release of VEGF and bFGF factors. Therefore, paracrine factors secreted by MSCs may have pivotal functions throughout the neovascularization process. The role of various secretory factors in the neovascularization process will be discussed below.

## Involvement of MSCs in the Neovascularization Process

**Figure 1:**
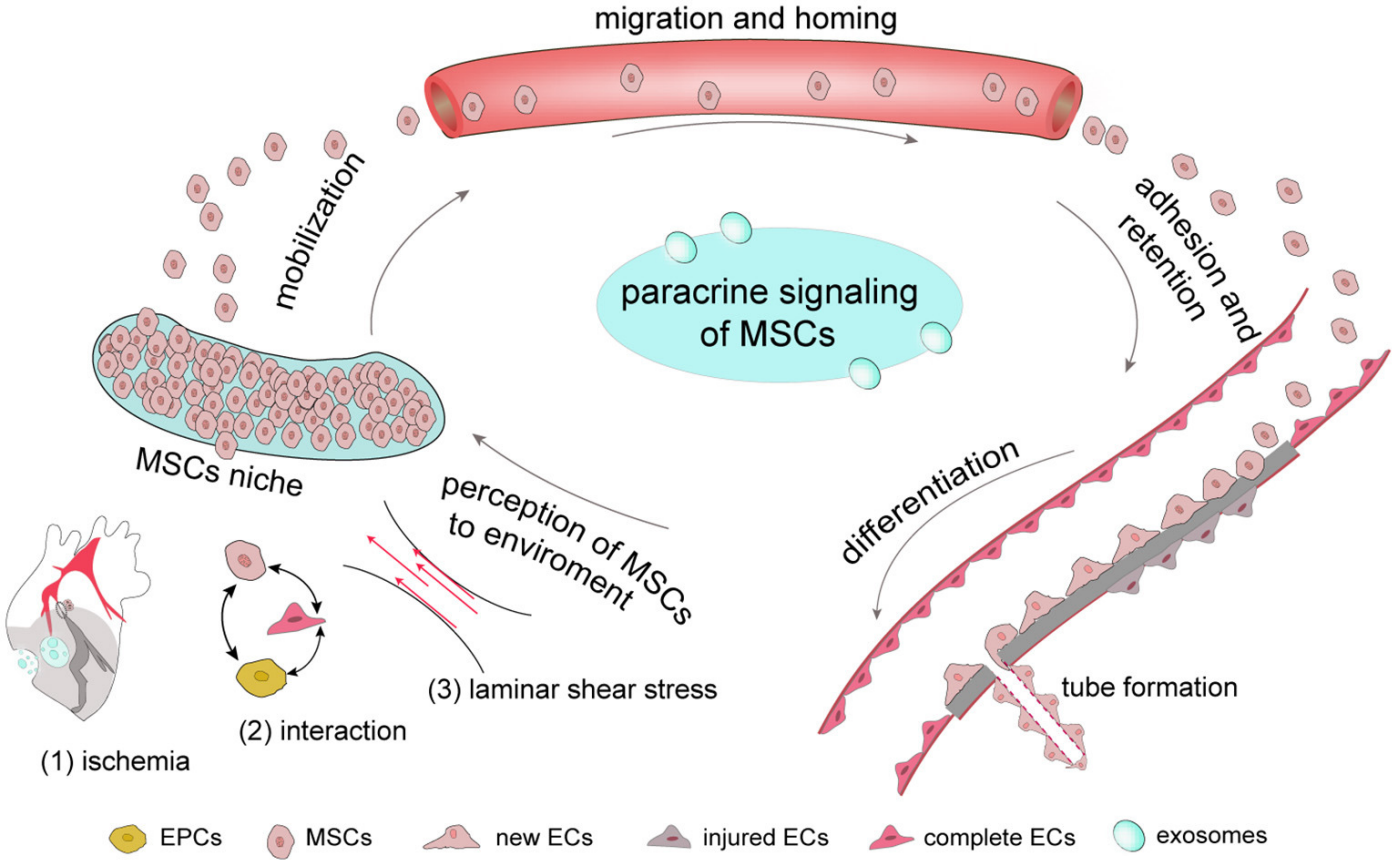
The complete process by which MSCs respond to ischemia In general, MSCs are stored in their niches, which retain adult stem cell in a dormant state. Once tissue is damaged, signals, including those involve in ischemia-associated pathways, cell-cell interaction and stress mobilize stem cells to migrate from the stem-cell niche to damaged tissues, where they adhere, self-renew and differentiate. Once ischemia occurs, MSCs have the ability to secrete a number of growth factors through their paracrine function to promote new tube formation of ECs to provide new blood for ischemic tissue.

The process by which MSCs promote neovascularization involves in a number of steps. First, once ischemia occurs which also follows stress change, MSCs can perceive the associated changes and are mobilized from their niches to migrate and adhere to ischemic tissue to proliferate and differentiate. Notably, MSCs secrete various factors, including chemokines and growth factors, and this paracrine function is carried out throughout the neovascularization process (Figure 1). The completion of all biological processes depends on the cooperation of different types of cells, and the neovascularization process requires the collaboration of ECs, endothelial progenitor cells (EPCs) and pericytes. In addition, exosomes derived from MSCs act as a messenger that participate in cell-to-cell communication.

### Environmental Perception by MSCs

#### Perception of Hypoxia by MSCs

Despite the benefits of MSC transplantation in cardiac tissue, detailed *in vivo* observations have shown that MSCs only survive for a brief period after engraftment due to harsh microenvironmental conditions (including ischemia, inflammation and anoikis) in the infarcted myocardium (34). However, this environment contributes to the mobilization of MSCs from their niches.

MSCs originated from the bone marrow microenvironmental niche exhibit low oxygen tension. O_2_ is a necessary factor in the maintenance of cell life as the final receptor in the intracellular aerobic respiration electron transport chain and is a substrate of some enzymes. Once the supply of O_2_ is insufficient, the hypoxia signal will be rapidly transmitted to nucleus and initiate related gene expression to maintain oxygen homeostasis and the balance of energy metabolism between the cells and organism. Hypoxia inducible factor 1 (HIF-1), which has a dimeric complex composed of HIF-1a and HIF-b subunits, is oxygen-sensitive and the most important transcription factor affecting gene regulation under hypoxia (35). Once ischemia occurs, HIF-1 increases the expression of angiogenesis-associated genes, including VEGF, its receptors Flk-1 and Flt-1, bFGF and the fibrinogen system (36, 37). At the same time, HIF-1 improves the expression of proteases, such as membrane type matrix metalloproteinases, which hydrolyzes extracellular protein to promote cell migration, matrix reconstruction and the formation of tubule-like structures (38).

Hypoxia is also a basic aspect of the microenvironment that determines the differentiation of MSCs. Compared with a normoxia group, VEGF expression in embryonic and MSCs under hypoxia was observed to be significantly increased (39–41). Likewise, the *in vivo* administration of hypoxia-inducible VEGF-engineered MSCs was shown to induce ischemia-responsive VEGF production and lead to a significant increase in myocardial neovascularization after myocardial infarction in rats (39).

#### Cell-Cell Interactions

In 1997, Asahara et al. (42) identified and named a small population of CD34^+^ cells as “EC progenitors.” Indeed, EPCs are involved in a number of processes during angiogenesis, including mobilization, differentiation into ECs, homing, paracrine function and others (43, 44). Coculture of EPCs and MSCs significantly increased the transcription levels of endothelial specific markers, including vWF, CD31, VE-cadherin, Flk-1 and Flt-1 (45) and enhanced tube-like formation (46) through platelet derived growth factor (PDGF), Notch and TACE/TNF alpha signaling (45, 47). Joensuu et al. (48) noted that in cocultures of human MSCs and peripheral blood mononuclear cells, the previously nonadherent cells attached and started to elongate and form tube-like structures within 1 week concomitant with VEGFR1 upregulation, and platelet endothelial cell adhesion molecule 1 (PECAM-1) and endoglin-positive vessel-like structures were observed after 20 days. In addition, MSC-EC interactions were observed to decrease endothelial permeability induced by lipopolysaccharide through hepatocyte growth factor (HGF) by restoring the integrity of endothelial monolayers and remodeling endothelial intercellular junctions (49). VEGF secreted by stem cells from apical papilla is also used by human umbilical vein endothelial cells to increase the number of endothelial tubules, tubule lengths, and branching points (50).

#### Laminar Shear Stress and Pulsatile Stress

There are many force-sensitive molecules on the cell surface, such as cilia, integrins, ion channels and plaque proteins. Integrins connect the cytoskeleton and extracellular matrix through adhesive plaque and transform the force signals into intracellular biological signals through this plaque (51, 52). Considering the key role of shear force in the differentiation of ECs, researchers reported that such mechanical stimulation in cell culture *in vitro* was equally effective for the differentiation of stem cells into ECs (53). MSCs are highly reactive to mechanical stimuli in the environment, and different types of stress on the same MSC population will lead to different differentiation results (54). After generating canine BMSCs under shear stress provided by a pulsatile bioreactor for 4 days, the expression of endothelial cell markers, such as PECAM-1, VE-cadherin and CD34 was observed to be significantly increased (55). Fisher et al. (56) noted that AMSCs could form cords but failed to take up acetylated low density lipoprotein (acLDL) or express molecular markers after being cultured in endothelial cell growth supplement. Only the subsequent exposure of stem cells to shear stress did the cells exhibit realignment, acLDL uptake and CD31expression, indicating that stem cells differentiation to ECs requires the synergism of biochemical and shear force.

### Dynamic Process of MSCs to Repair Ischemic Tissue

#### Mobilization

MSC mobilization is key for its involvement in tissue repair following their sensing of hypoxia, stress or other signals. An anoxic environment is one of the factors that induces stem cells to migrate out of their niches. Prolyl hydroxylase (PHD) and factor inhibiting HIF-1 (FIH) are key oxygen sensors in MSCs. HIF-1α upregulation by double knockdown of PHD and FIH synergistically increases stem cell mobilization and myocardial angiogenesis and improves cardiac function (57). The high concentration of growth factors outside of stem-cell niches may be another factor causing MSCs to mobilize from their original niches. Stromal cell-derived factor-1 (SDF-1 α)/Cxc chemokine receptor 4 (CXCR-4) are part of the most important chemotactic axis regulating MSC mobilization and migration. VEGF and insulin-like growth factor-1 (IGF-1)-overexpressing MSCs accelerate BMSC mobilization via the activation of SDF-1 α/CXCR4 signaling to promote myocardial repair (58, 59). Wan et al. (60) showed that active transforming growth factor β (TGF-β) also control the mobilization and recruitment of MSCs to participate in vascular repair. In addition, high-intensity exercise may be a potent stimulus that promotes circulating mesenchymal cells mobilization in patients with stable coronary artery disease (61).

#### Migration and Homing of MSCs

Homing and migration comprise a key step after MSC mobilization. Microenvironmental interactions between hypoxia and MSCs may control the ability of MSCs to migrate and their migration direction. In hypoxic tissue, SDF-1 and CXCR-4 are also important factors for cell migration. Ischemic myocardial and vascular tissues secrete SDF-1 to attract CXCR-4-expressing cells, particularly their therapeutic progenitors. Yu et al. (62) showed that SDF-1/CXCR-4 may mediate the migration of BMSCs toward heart MI through activation of PI3K/Akt signaling. Growth factors play an important role in the process of MSC migration. The stimulation of SDF-1α expression in infarcted hearts by VEGF-overexpressing MSCs was observed to result in the massive mobilization and homing of BMSCs (59). TGF-β1, HGF, IGF-1 and endothelial nitric oxide synthase (eNOS) also promoted the migration and homing BMSCs to the ischemic myocardium (63–65). Schmidt et al. (31) showed that low concentrations of bFGF attracted cells, indicating that bFGF may direct the migration of MSCs. In addition, Yan et al. (66) observed that C1q/tumor necrosis factor-related protein-9 (CTRP9) enhances AMSC proliferation and migration through the ERK1/2-MMP-9 signaling pathway and also promotes anti-apoptotic/cell survival via ERK-Nrf2/anti-oxidative protein expression. MiRNA, like miR-206 also involved in migration of MSCs by targeting Pim-1 (67).

#### Proliferation and Survival

Although MSCs transplantation is a promising therapeutic approach for IHD, the low viability of MSCs after transplantation needs to be improved. Hypoxic preconditioning may improve the functional survival and therapeutic efficiencies of engrafted BMSCs, at least in part through autophagy regulation (68). Some growth factors, including increased VEGF, TGF-β, IGF-1, SDF-1a and angiogenin were shown to enhance MSC survival and vasculogenesis in an MI model (69). Preconditioning with other factors, such as protein kinase C epsilon (εPKC), CTRP9, dimethyloxalylglycine and connexin-43 improves the retention and survival of transplanted MSCs in rat MI through the SDF-1/CXC and PI3K/AKT pathways (66, 70–72). Qu et al. (73) showed that atorvastatin, a hypolipidemic agent, has a protective effect on cardiomyocytes against apoptotic cell death in infarct and peri-infarct areas and could also increase the survival rate of implanted BMSCs in acute myocardial ischemia.

#### Adhesion and Retention

MSCs need to stay and adhere to ischemic tissue to play their important role in ischemic tissue repair. Molecular imaging studies have shown that <5% of MSCs engraft in ischemic tissues after being intravenously injected, with most of them dying within few hours after administration (74). This poor engraftment may be attributed to the constant blood flow and the harsh environmental conditions after acute ischemic injury. Since the long-term efficacy of cell therapy is proportional to the number of retained cells, this low retention and viability needs to be improved. Increasing the ability of MSCs to adhere to the ischemic tissues is key to improving their retention and viability. IGF-1 can increase the adhesion of MSCs and prolong their survival under hypoxia *in vitro* through PI3K activation (75). MSC adhesion can also be promoted by increasing the expression of integrin-linked kinase, periostin, and 2, 4-dinitrophenol (76–78). Reactive oxygen species (ROS) inhibit the cellular adhesion of engrafted MSCs, indicating that the elimination of ROS may be a novel strategy for improving the survival of engrafted MSCs (79). Bortolotti et al. (80) demonstrated that cardiotrophin-1 (CTF1) increases the retention and adhesion of BMMSCs to protect BMSCs from apoptosis, identifying it as a new powerful cytokine promoting cell engraftment. The retention and survival of transplanted MSCs was also shown to be improved by the overexpression of εPKC in AMI rats through the SDF-1/CXC and PI3K/AKT pathways (70).

## Interactions Among MSCs and ECs, EPCs and Pericytes

**Figure 2:**
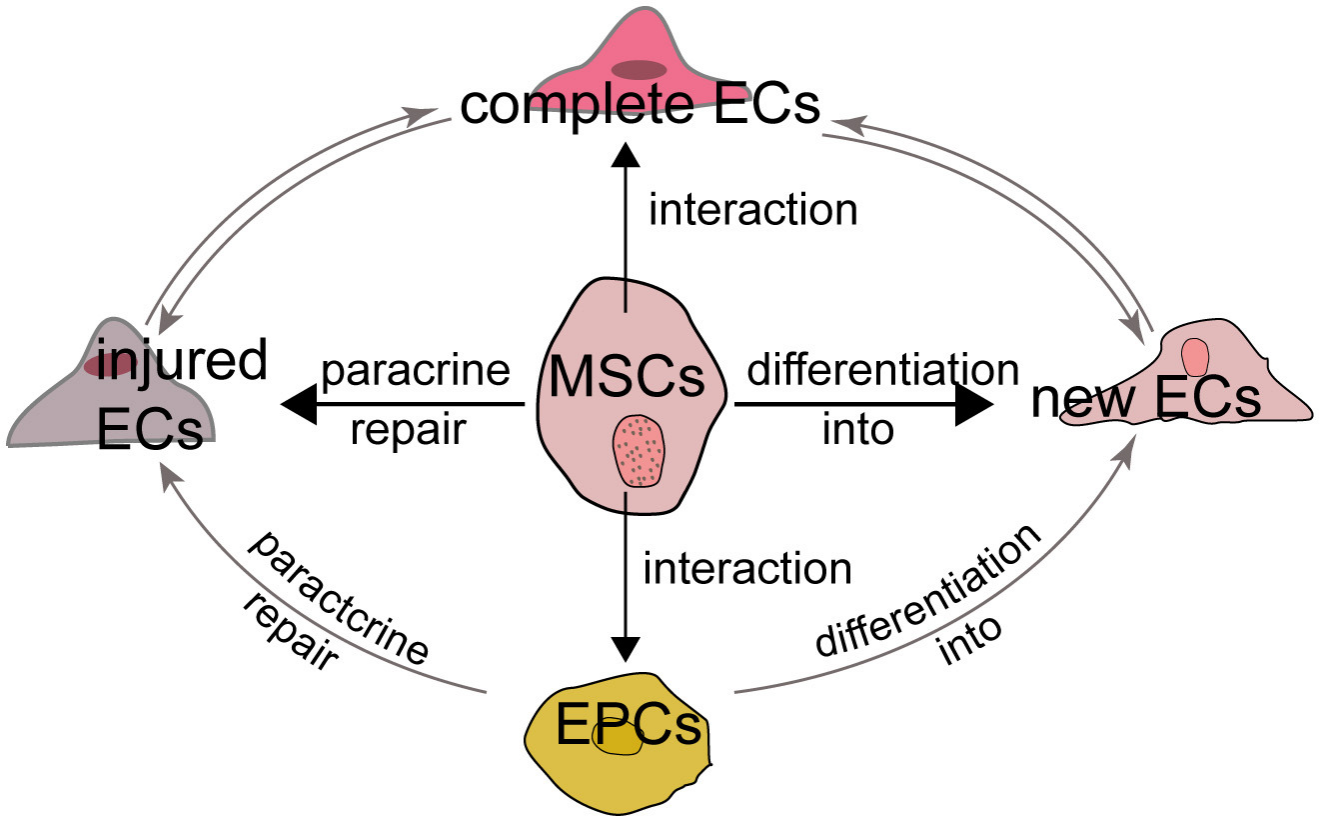
MSCs and endothelial cells The relationship between MSCs and ECs can be summarized as follows. First, MSCs can repair the injured but not dead ECs through their paracrine function to induce the release of growth factors. Second, MSCs have the potential to differentiate into new ECs although it is controversial. Third, the interaction and crosstalk between MSCs and existing ECs promotes the formation of new ECs and the repair of injured ECs. Last, MSCs also interact with EPCs to influence EC formation and function.

Multiple cell types are known to be involved in the processes of angiogenesis and vasculogenesis, including MSCs, ECs, EPCs and pericytes. In particular, ECs are indispensable for angiogenesis and the relationship between MSCs and ECs mainly attribute to the following aspects. First, MSCs secret growth factors which can repair the injured but not dead ECs through their paracrine function. Second, the interaction and crosstalk between MSCs and existing ECs promotes the formation of new ECs and the repair of injured ECs. Third, MSCs have the potential to differentiate into new ECs although it is controversial. Last, MSCs also interact with EPCs and pericytes to influence EC formation and function (Figure 2).

The ability of MSCs to limit infarct size may attributed to their pro-angiogenesis activity through existing ECs (Figure 2). The stimulated angiogenic activity of ECs is associated with the secretion of various growth factors and cytokines, including VEGF, HGF, IL-6, TGF-β1 and monocyte chemoattractant protein-1 (81). Lu et al. (82) showed that nestin(+) BMSC transplantation improved cardiac function in a mouse AMI model by recruiting resident cardiac ECs to the infarcted border region. BMSCs also rescued injured ECs through modulation of mitophagy or activation of signaling pathways such as PI-3K/AKT/m-TOR/eNOS and p38/MAPK (83, 84). Hypoxia also influences the interactions between the endothelium and MSCs (85).

In addition to the interactions between MSCs and ECs, studies showed MSCs had the ability to differentiate into ECs to promote angiogenesis in some degree although it was still controversial. For example, Otto et al. (86) did not observe MSC transdifferentiation into cardiomyocytes, ECs or SMCs and that the transdifferentiation of MSCs into cardiomyocytes or vascular cells did not significantly contribute to the improvement of cardiac function. Conversely, Silva et al. (87) showed that BMSCs promoted the angiogenesis of dog ischemic myocardium by differentiating into ECs, which accelerated the establishment of collateral circulation. Studies support MSCs own the potential to differentiated into ECs according to the below reasons. First, MSCs are multipotent stem cells derived from the mesoderm. Theoretically, MSCs can be differentiated into all mesoderm derived cells, and since ECs are mesoderm-derived cells, MSCs have the potential to differentiate into ECs. Second, MSCs express molecular markers of early ECs, such as VEGF receptor 2 (VEGFR-2/Flk-1/KDR) and bFGF, indicating that ECs can be derived from mesenchymal colonies and that MSCs arise from precursors with angiogenic potential (31, 88). Last, a series of *in vivo* and *in vitro* experiments proved that MSCs can differentiate into ECs. Oswald et al. (10) successfully used 2% fetal bovine serum supplemented with 50 ng/mL VEGF to induce BMSCs to differentiate into ECs *in vitro*. They observed that differentiated cells increased the expression of endothelial-specific markers, such as KDR and VEGF receptor 1 (VEGFR-1/Flt-1), and formed capillary-like structures. Furthermore, the process of MSC differentiation into ECs may require the synergy of bFGF, IGF, epidermal growth factor (EGF) (89, 90). ERK signaling may also involve in the differentiation of porcine AMSCs into ECs (90).

Furthermore, MSCs also function with EPCs to promote tissue repair. As a precursor of ECs, EPCs also differentiate into ECs and promote ischemia angiogenesis through their paracrine function (91, 92). MSCs could attract and promote the migration and vascularization of EPCs, which may depend on a positive feedback loop between CXCR-2 and CXCR-4 (93, 94). The viability and ability of MSCs to promote nerve regeneration is also improved by EPCs through PDGF-BB/PDGFR-β signaling (95). Rossi et al. (96) found MSCs and EPCs into the hind limbs of ischemia model together accelerated ischemic muscle recovery through an endoglin-dependent mechanism. Consequently, MSCs, ECs and EPCs may have a synergistic effect in ischemic tissue repair (Figure 2).

Pericytes, also known as mural cells, wrap around ECs in arterioles, capillaries and venules to regulate the maturation of ECs, stabilize the microvascular wall and promote angiogenesis. Although pericytes are surrounded by a basement membrane, they contact the ECs with through a “peg and socket” mechanism through holes in the basement membrane. Studies have shown that pericytes also communicate with ECs via paracrine signaling to improve tissue repair (97, 98). It is notable that pericytes also have stem cell-like properties and exhibit the morphology, mitotic activity and surface antigens of MSCs (99) and are seemingly able to differentiate into adipocytes, chondrocytes, osteoblasts, neurons, astrocytes and oligodendrocytes, leading them to be identified as MSCs (100–102). However, it is still debated whether pericytes are MSCs. Guimaraes-Camboa et al. (103) challenged this concept and suggested that mural cells do not intrinsically behave as MSCs during aging and repair in multiple adult organs using a transgenic cell line. Over 2 years, the study showed that Tbx18 lineage-derived cells maintained their perivascular identity in the brain, heart, muscle and fat, indicating that mural cells do not exhibit an overt potential to give rise to other cell types. In contrast, MSCs can serve as a potential source of pericytes and induce vasculogenesis as mentioned previously (13, 104, 105), but similar to the multi-differentiation potential of MSCs, there needs to be standard guidelines for assessing pericyte differentiation in future studies. Furthermore, MSCs secrete various growth factors, including PDGF, which serves as a biomarker and crucial factor controlling the differentiation and recruitment of pericytes (106–108). These findings indicate that MSCs may regulate the recruitment of pericytes to injured tissue to participate in angiogenesis, but the associated mechanisms between MSCs and pericytes need to be further elucidated.

## MSCs and MSC-Derived Exosomes

Exosomes are a type of extracellular microvesicle secreted by multiple eukaryotes. Compared with cell therapy, MSC-derived exosomes (MSC-exos) have lower immunogenicity and are safer and more efficient, providing a new strategy for tissue regeneration via cell-free therapy (109, 110). MSC-exos are a type of message carrier that harbor a modifiable content of microRNAs, mRNAs and proteins, mediating communication between cells and functioning as key mediators of the paracrine effect of MSCs (111, 112). The pro-angiogenesis function of MSC-exos has been demonstrated in a number of studies. For instance, exosomes from MSCs overexpressing Akt, HIF-1α or CXCR-4 were shown to accelerate EC proliferation, migration and tube-like structure formation *in vitro*, as well as blood vessel formation to improve cardiac function in an MI model (113–115). MSC-exosomes may also have anti-inflammatory activities in MI model (116). Currently, the application of MSC-exos primarily focuses on preclinical experiments. One of the key problems for exosome clinical therapy is how to collect and purify enough exosomes so that they can be used safely. Andriolo et al. (117) developed a GMP-class method for the mass preparation of stem cell-derived exosomes to enable them to be used in future clinical applications. Indeed, as they are secreted by MSCs, MSC-exos have similar biological properties to MSCs to some extent. MSC-exos also have paracrine functions and mediate communication between MSCs and ECs, and they are also influenced by microenvironmental stress conditions, such as hypoxia and irradiation (118, 119). Furthermore, as they harbor a part of and not the entirety of MSC contents, MSC-exos are not an MSC “mini-me” and cannot replace MSCs in some respects, including their multiple differentiation and proliferation abilities.

## Application of MSCs in IHD Clinical and Preclinical Practice

The results of numerous clinical and preclinical studies have indicated that MSC transplantation is safe, significantly improves cardiac function and decreases infarct size and fibrosis in ischemic patients, which may be associated with the survival, retention, angiogenesis, paracrine action and the anti-apoptosis activities of MSCs (Tables 2, 3). Although cellular therapies hold great promise for the treatment of human IHD and have good safety, the efficacy of MSCs remains disputable, especially when used in clinical trials. Meta-analyses of randomized clinical trials showed that the transplantation of BMSCs resulted in limited improvement on cardiac function for MI patients (171, 172). As it was showed in Table 2, some clinical trials proved MSC transplantation did not improve LVEF although it may limit infarct size. Different from clinical trials, MSC transplantation in animal experiments showed significantly elevated LVEF in most studies (Table 3).

**Table 2:** Completed clinical trials using MSCs to treat ischemic cardiovascular diseases registered at clinicaltrials.gov.

**NCT**	**Conditions**	**Interventions**	**Phase**	**Patients No/follow-up**	**Endpoint**	**LVEF improved**	**Study type**	**Cell quantity**	**Method**	**Data**	**References**
NCT00950274	MI	CD133^+^ ABMSCs	III	82/6 mon	LVEF, LVEDV, LVESV, scar size, LV mass, NT-proBNP	Yes	R,PA,D	0.5-5x10^6^	IM	2009.7–2016.3	(120)
NCT00669227	AMI	ABMCs	II	42/6 mon	LVEF, LVEDV, LVESV, infarct size	No	R,PA,D	381 × 10^6^	ICA	2005.10–2009.1	(121)
				42/36 mon	LVEF, LVEDV, LVESV, infarct size, MO	Yes	R,PA,D	324 × 10^6^			(122)
NCT00893360	MI/LVD	Autologous cardiosphere-derived stem cells	I	25/6 mon	Safety, LVEF, scar mass, systolic wall thickening	No	R,PA,N	12.5–25 × 10^6^	ICA	2009.5–2012.2	(123)
				25/13.4 mon	Safety, LVEF, LVEDV, LVESV, scar mass, scar size,	Yes					(124)
NCT01033617	MI/HF	ABMCs(CD133^+^)	–	40/ 6 mon	LVEF, LVEDV,LVESV,	No	R,PA,Q	0.5–10 × 10^6^	IM	2009.10–2016.6	(125)
NCT00279175	AMI	ABMCs	III	204/12 mon	LVEF, safety	Yes	R,PA,D	236 × 10^6^	ICA	2004.4–2010.10	(126, 127)
NCT00114452	MI	Allogeneic hMSCs	I	53/6 mon	Safety, LVEF,LV remodeling	Yes	R,PA,D	0.5, 1.6, 5.0 × 10^6^/kg	ICV	2005.2–2009.2	(128)
NCT01291329	STEMI	Human umbilical WJ-MSC	II	116/18 mon	Safety, LVEF, perfusion,	Yes	R,PA,Q	6 × 10^6^	ICA	2011.2–2012.7	(129)
NCT00883727	MI	Allogeneic BMSCs	I/II	20/2 year	Safety, LVEF, perfusion, infarct volume	No	R,PA, D	2 × 10^6^/kg	ICV	2009.4–2012.8	(130)
NCT00765453	MI	ABMCs	II	100/12 mon	LVEF, infarct size, NT-proBNP	No	R,PA,T	59.8 (1.9CD34^+^) × 10^6^	ICA	2008.3–2018.3	(131)
NCT00264316	STEMI	ABMCs	II	67/4 mon	LVEF, LVEDV, LVESV, infarct size, systolic wall thickening	No	R,SA,D	304 (72MNCs) × 10^6^	ICA	2003.5–2005.12	(132)
NCT00199823	AMI	ABMMCs	II	100/12 mon	Prothrombotic markers, LV function	No	R,PA,S	68 × 10^6^ (0.7 × 10^6^ CD34^+^)	ICA	2003.9–2006.5	(133, 134)
NCT00313339	STEMI	ABMSC CD34+	I	31/6 mon	Safety, LVEDV, LVESV, LVEF, infarct size, perfusion	No	R,FA,N	5, 10, 15 × 10^6^CD34^+^ cells	ICA	2006.3–2013.3	(135)
NCT00684060	IHD /LVD	ABMSC	II	80/6 mon	LVEF, wall motion, LV volumes, infarct size, relationship of Ratio of CD133^+^,CD34^+^ cells on LVEF	Yes	R,PA,D	150 × 10^6^	ICA	2008.7–2012.2	(136–138)
NCT00684021	STEMI/ LVD	ABMSC	II	120/6 mon	Safety, LVEF, wall motion, LV function	No	R,PA,D	150 × 10^6^	ICA	2008.7–2012.11	(139)
				95/12 mon	LVEF,LV volumes, infarct size	No					(140)
NCT00355186	STEMI	ABM-MNCs	II	200/4 mon	LVEF, LVEDV, LVESV	No	R,FA,N	153 (119) × 10^6^	ICA	2006.8–2012.11	(141)
				200/ 12 mon	LVEF, LV volumes, scar size, N-BNP	No					(142)
NCT01167751	MI	BM-MNCs; CD133^+^ cells	II/III	90/6,18 mon	Safety, LVEF, systolic wall thickening	Yes	R,PA,D	MNCs:564.63 × 10^6^; CD133^+^cells:8.19 × 10^6^	IM	2008.1–2012.7	(143)
NCT00268307	AMI	ABMCs	I	40/6 mon	LVEF, LVEDV	Yes	R, CA, D	100 × 10^6^	ICA	2005.12–2010.9	(144)
NCT02439398	AMI	Allogeneic hCSCs	I/II	49/12 mon	Safety, infarct size	–	R,PA,D	35 × 10^6^	ICA	2014.6–2016.11	(145)
NCT00395811	CABG	ABMMNC	I/II	60/6 mon	safety, LVEF, LVEDV, LVESV,	Yes	R,PA,D	100 × 10^6^	Graft	2007.1–2009.6	(146)
NCT00418418	Ischemic HF	ABMMC	II	39/12 mon	LVEF, LVEDV, LVESV, infarct size, safety,	No	R,FA,Q	5–1,000 × 10^6^	ICA	2006.10–2010.12	(147, 148)
NCT00363324	STEMI	BMSCs	II/III	80/6 mon	LVEF, LVEDV, LVESV	Yes	R,PA,D	360(2.6 CD34+)x10^6^	ICA	2005.1-2009.11	(149, 150)
NCT01495364	STEMI	CD34+	II	161/ 12 mon	Safety, LVEF, infarct size,	No	R,PA,D	10x10^6^±20%	ICA	2011.12-2016.4	(151)
NCT00289822	IHD	BMCs/CPCs	II	75/ 3 mon	LVEF	Yes	R,CA,N	BMCs: 205 × 10^6^;CPCs:22 × 10^6^	ICA	2002.1–2005.1	(152)
NCT01234181	MI	Hypoxia BMSCs	–	34/1 year	Safety, LVEF, LVEDV, LVESV, wall motion, perfusion	No	R,PA,N	10 × 10^6^	ICA	2010.11–2012.12	(153)
NCT01076920	CMI/LVD	ABMSCs	I/II	10/2 year	Safety, LVEF, myocardial viability and contraction	Yes	SA;N	61.5 × 10^6^	IM	2009.10–2014.9	(154)
NCT01087996	ICM	BMSCs	I/II	30/13 mon	Safety, LVEF, LVEDV, LVESV, immunologic monitoring, quality of life, pulmonary function	–	R,PA,N	20, 100, 200 × 10^6^	TE	2010.4–2011.9	(155)

*R, Randomized; PA, Parallel Assignment; FA, Factorial Assignment; CA, Crossover Assignment; SA, Single Group Assignment; DR, Double Randomized Controlled Trial; Q, Quadruple (Participant, Care Provider, Investigator, Outcomes Assessor); T, Triple (Participant, Care Provider, Investigator); D, Double blind; S, Single (Outcomes Assessor); N, None (Open Label); ABMMCs, Autologous Bone Marrow Mononuclear Cells; ABMCs, autologous bone-marrow cells; hCSCs, human cardiac stem cells; LVEDV, Left ventricular end-diastolic volume; LVESV, left ventricular end-systolic volume; MO, microvascular obstruction; IHD, ischemic heart disease; LVD, left ventricular dysfunction; ICM, ischemic cardiomyopathy*.

**Table 3:** Representative animal studies performed using MSCs in ischemic models.

**Disease model**	**Animal source**	**cell source/type**	**Dose**	**Method/No. of sites**	**Treatment/end time**	**Preclinical outcome**	**Mechanisms**	**Specific treatment**	**References**
MI/IR	Mice	mice/ADSCs	2 × 10^5^	IM/3	0 h/-	↑LVEF, ↓fibrosis	↑ADSCs survival/retention, migration, angiogenesis; ↑cardiomyocyte adhesion, proliferation; ↑MMP-10/13 and/or HGF	N-cadherin	(156)
IR	Adult male SD rats	SD rat within 6–7 d/ ADSCs	2 × 10^6^	Iv/-	0, 24 h/24 h,72 h,28 d	↑LVEF, ↓infarct size	↓Neutrophil number by enhanced M2 macrophage and macrophage efferocytosis	–	(157)
IR	Female Gottingen mini-swine	Male Gottingen mini-swine/Cortical bone stem cells	2 × 10^7^	IM/-	1.5–2 h/3 d, 7 d, 3 mon	↑LVEF, ↓infarct size	↑Macrophage and T-cell populations; ↓cardiomyocyte apoptosis	–	(158, 159)
MI	Male C57 mice	Human umbilical cord blood-MSCs	2 × 10^5^	IM/4	0/-	Protects cardiac function (↓infarct size)	↓Apoptosis and autophagy of myocardial cells; ↑tube formation of ECs	Exo-SDF1	(160)
MI	Male C57/BL6 mice	-/MSCs	2 × 10^5^	IM/5	30 min/1, 28 d	↑LVEF, ↓LVEDV, LVESV, scar size,	↑Autophagic flux through exosome containing mainly miR-125b-5p	Exo- MSCs	(161)
IR MI	Female Göttingen swine	Male Yorkshire swine MSCs/CSCs	200 × 10^6^/1 × 10^6^	TE/10	-/3 mon	↑LVEF, perfusion, ↓LVEDV, LVESV, scar size, remodeling	↑Cardiac regeneration	–	(162)
MI	C57/BL6 mice; CTRP9 knock-out mice	EGFP-TG mice with C57BL/6J background/ADSCs	1 × 10^5^	IM/3	1, 3, 7, 14 d/3 d, 4 w	↑LVEF, fibrotic area	↑Proliferation/migration by ERK1/2-MMP-9, ↓poptotic /oxidant via ERK-Nrf2	CTRP9	(66)
MI	C57/BL6 Mice	male C57/BL6/BM-MSCs, ATMSCs	2 × 10^5^	IM/1	-/IM after 1, 7, 14, and 21 d, LV after 21, 60 d	↑LVEF, LVFS	↑BM-MSC adhesion, ↓apoptosis, ↑focal adhesion kinase	CTF1	(80)
I/R	Female Large White pigs	male Large White pigs/ATMSCs	10 × 10^6^	ICA/-	15 min after reperfusion/2 days, 60 d	↑Myocardial perfusion (vascular density), not LV Function	Pro-angiogenic factors: VEGF,SDF-1a, GM-CSF; Anti-apoptotic, inflammatory and collagen deposition	–	(163)
MI	SD rats	Aged and young male hMSCs	10 × 10^6^	IM/5	30 min/28 d	↑LVEF, ↓fibrosis/scar size	↑Angiogenesis/ survival; against apoptosis	SRT1720	(164)
MI	C57BL/6 mice	Synthaetic hBMSCS	1 × 10^5^	IM/-	0/15 d	Mitigated LV remodeling	↑Angiogenesis	–	(165)
MI	Male SD Rats	SD rats /BMSCs	22 × 10^6^	Tail vein	2 d/IF: weeks 1, 2, 4; LV: 3 d, 1,3,6 w	↑Cardiac function (LVEF, LVDD, LVSD)	↑Angiogenesis by the tropism of MSCs to the MI area through SDF-1	VEGF-encapsulated	(166)
AMI	Female Large White pigs	Pigs /ATMSCs	50 × 10^6^	IM /7-8	0/2, 15, 30 d	Cardiac function not significantly improved (LVEF)	↓Inflammation, ↑angiogenic process	IGF-1 /HGF	(167)
MI	Male Corriedale sheep	Corriedale male sheep/ BMSCs	20 × 10^6^	IM/-	30 min/30, 60 d	↓Infarct volume; ↑LVEF	↑Angio-/arteriogenesis, ↓apoptosis by HIF1-mediated overexpression of EPO, iNOS, VEGF, and ANG-1	HIF1-a	(168)
CMI	Female pigs	Human umbilical cord-derived MSCs	30 × 10^6^	ICA/ICV/-	4, 5, 6 w/ 4 w	↑LVEF, perfusion; ↓apoptosis, fibrosis	↑Angiogenesis by VEGF and Ang	–	(169)
MI	Male Cynomolgus monkeys	Cynomolgus monkey/BMSCs	10 × 10^6^	IM/5	0/3, 28, 90, 180, 270 d	↑LV function, ↓infarct size	↑Cardiomyocyte proliferation, vascular density, myocardial glucose uptake, engraftment, paracrine activity(EPO, HIF1-α, ANG-1); ↓endogenous cell apoptosis	Hypoxia	(170)
AMI	SD rat	Male SD rat/BMMSCs	1 × 10^6^	IM/5	0/1 day, 4 w	↑LVEF, ↓LVEDD, LVESD), remodeling improved	↑Retentionl:SDF-1/CXC and PI3K/AKT; ↑survival:VEGF, bFGF, TGFβ, cTnI, vWF, SMA and factor VIII	PKCε	(70)

*ICA, intra-coronary artery infusion; ICV, intra-coronary vein infusion; IM, intramyocardial; TE, transendocardial; ↑, indicators increased or improved; ↓, indicators decreased or worsened*.

Clinical patients are different from animal ischemia models, and the efficacy of MSCs in clinical practice is influenced by many different factors, such as (1) disease etiology and severity of patients, and (2) the type, number, delivery route and time, retention, survival, proliferation and differentiation of MSCs. Another meta-analysis showed that MSCs are more effective in patients with lower baseline left ventricular ejection fraction (LVEF) (≤50%), and the effects of cells that were transferred at 3–7 days post-AMI was superior to those transferred within 24 h or more than 7 days in improving LVEF and decreasing LV end-systolic and diastolic dimensions (173), which suggested transplantation time was a key factor to influence cardiac function. Compared with clinical trials, animal experiments are easier to obtain positive results because of their simplicity, such as the MI model can be established uniformly by ligation of the left anterior descending coronary artery. Compared with MSCs intervention alone, pretreated MSCs with some growth factors together may get more efficacy (Table 3).

It is notable except for growth factors, more attention has been paid to natural botanical medicines. EGb761, an extract of *Ginkgo biloba*, was shown to exhibit a biphasic effect on hypoxia/serum deprivation-induced BMSC apoptosis, and its effect was closely associated with the PI3K/Akt and caspase-9 signaling pathways (174). *Salvia miltiorrhiza* is a widely used traditional Chinese medicine in cardiovascular diseases, and its constituent Tanshinone IIA was observed to decrease infarct size by increasing the recruitment of BMSCs to the infarct region by upregulating the SDF-1/CXCR-4 axis in a rat MI model (175). In addition to botanical medicines, chemicals such as statins, as the most commonly used lipid-lowering agents, exert activity toward a wide spectrum of cellular functions in addition to their lipid-lowering effects, including anti-inflammatory, anti-apoptotic, anti-fibrotic and pro-angiogenesis effects (176, 177). The results of multiple studies have suggested that atorvastatin has the ability to increase the survival rate of implanted BMSCs in an MI model, and combined with MSCs, it also ameliorated the cardiac milieu by reducing inflammatory cell infiltration, myeloperoxidase activity and cardiac fibrosis (178, 179). Furthermore, activation of the JAK-STAT pathway may play important role in the ability of rosuvastatin to increase the efficacy of transplanted MSCs (178). Other factors, including glucagon-like peptide-1-eluting (GLP-1), lipopolysaccharide and lysophosphatidic acid also exert pro-angiogenesis effect by promoting the enhanced expression of cytokines and growth factors (180–183).

## Strategies and Future Directions

MSCs display robust reparative properties through their paracrine and differentiation abilities that can limit apoptosis, enhance neovascularization and direct positive tissue remodeling. However, some problems with MSCs remain and must be solved before they can have widespread use. MSCs are important infiltrating cells that are also drived by blood and vasoconstriction. So the first problem is the low survival and retention of transplanted cells *in vivo* which limits their overall effectiveness in clinical usage. Consequently, identifying strategies to improve cell survival and retention *in vivo* is a priority. However, cell transplantation is affected by many factors, each of which may have an impact on the survival of transplanted cells, and there is still no consistent recommendation for each factor. The microenvironment of transplanted cells directly affects the survival of stem cells. The blood supply in the marginal area of myocardial infarction is well known to directly affect the survival rate and recovery of cardiac function after cell transplantation. One important goal of cell transplantation is to promote angiogenesis in the ischemic area and reduce the generation of myocardial scars. Studies have shown that hypoxia-induced stem cells release a variety of factors to improve the microenvironment through anti-inflammatory and anti-fibrosis effects and by promoting angiogenesis (170). Hypoxia or other growth factors used to precondition stem cells may allow MSC survival and retention to be improved, but additional comparisons and a set of standards are needed to identify the most powerful factors.

Another important factor limiting the clinical application of stem cells is the shortage of effective monitoring methods for stem cells. The successful implementation of cell therapies requires a better understanding of cell fate after transplantation. Currently, there are three primary labeling methods for stem cells, including reporter genes, fluorescent dyes and nanoparticles, which require optical imaging, MRI and radionuclide imaging to trace the transplanted stem cells, respectively or in combination, with each technique having its advantages and disadvantages (74, 184). Thus, there is an urgent need to develop a nontoxic and noninvasive tracer technology that exhibits long term stability and that can also be used to dynamically monitor the survival status of transplanted cells with respect to processes such as migration and differentiation *in vivo*.

## Author Contributions

WS contributed to the design and manuscript writing. QX, RY, and YY collected and assembled data. WC contributed the review design and financial support. KC was responsible for proofreading and final approval of the review. All authors contributed to the article and approved the submitted version.

## Conflict of Interest

The authors declare that the research was conducted in the absence of any commercial or financial relationships that could be construed as a potential conflict of interest.
